# A network of stress-related genes regulates hypocotyl elongation downstream of selective auxin perception

**DOI:** 10.1093/plphys/kiab269

**Published:** 2021-06-12

**Authors:** Adeline Rigal, Siamsa M. Doyle, Andrés Ritter, Sara Raggi, Thomas Vain, José Antonio O’Brien, Alain Goossens, Laurens Pauwels, Stéphanie Robert

**Affiliations:** 1 Umeå Plant Science Centre, Department of Forest Genetics and Plant Physiology, Swedish University of Agricultural Sciences, 90183 Umeå, Sweden; 2 Department of Plant Biotechnology and Bioinformatics, Ghent University, 9052 Ghent, Belgium; 3 VIB Center for Plant Systems Biology, 9052 Ghent, Belgium; 4 Departamento de Genética Molecular y Microbiología, Facultad de Ciencias Biológicas, Santiago, 8331150, Chile; 5 Departamento de Fruticultura y Enología, Facultad de Agronomía e Ingeniería Forestal, Pontificia Universidad Católica de Chile, Avenida Libertador Bernardo O’Higgins 340, Santiago, 8331150, Chile

## Abstract

The plant hormone auxin, a master coordinator of development, regulates hypocotyl elongation during seedling growth. We previously identified the synthetic molecule RubNeddin 1 (RN1), which induces degradation of the AUXIN/INDOLE-3-ACETIC ACID (AUX/IAA) transcriptional repressors INDOLE-3-ACETIC ACID-INDUCIBLE3 (IAA3) and IAA7 in planta and strongly promotes hypocotyl elongation. In the present study, we show that despite the structural similarity of RN1 to the synthetic auxin 2,4-dichlorophenoxyacetic-acid (2,4-D), direct treatments with these compounds in Arabidopsis (*Arabidopsis thaliana*) result in distinct effects, possibly due to enhanced uptake of RN1 and low-level, chronic release of 2,4-D from RN1 in planta. We confirm RN1-induced hypocotyl elongation occurs via specific TRANSPORT INHIBITOR RESISTANT1 (TIR1)/AUXIN SIGNALING F-BOX (AFB) receptor-mediated auxin signaling involving TIR1, AFB2, and AFB5. Using a transcriptome profiling strategy and candidate gene approach, we identify the genes *ZINC FINGER OF ARABIDOPSIS THALIANA10* (*ZAT10*), *ARABIDOPSIS TOXICOS EN LEVADURA31* (*ATL31*), and *WRKY DNA-BINDING PROTEIN33* (*WRKY33*) as being rapidly upregulated by RN1, despite being downregulated by 2,4-D treatment. RN1-induced expression of these genes also occurs via TIR1/AFB-mediated auxin signaling. Our results suggest both hypocotyl elongation and transcription of these genes are induced by RN1 via the promoted degradation of the AUX/IAA transcriptional repressor IAA7. Moreover, these three genes, which are known to be stress-related, act in an inter-dependent transcriptional regulatory network controlling hypocotyl elongation. Together, our results suggest *ZAT10*, *ATL31*, and *WRKY33* take part in a common gene network regulating hypocotyl elongation in Arabidopsis downstream of a selective auxin perception module likely involving TIR1, AFB2, and AFB5 and inducing the degradation of IAA7.

## Teaser

A distinct inter-dependent transcriptional network of stress-related genes regulates hypocotyl elongation in Arabidopsis downstream of a selective auxin perception module.

## Introduction

Auxin is a master regulator of plant development, known to be involved in coordinating almost all aspects of plant growth (Strader and Zhao, 2016). Dissecting the regulation of specific features of auxin-coordinated plant development can therefore present challenges. Over the past few years, powerful chemical biology approaches have resulted in the isolation of synthetic auxin analogs, agonists, and antagonists displaying more potency and/or selectivity than auxin itself, which has greatly assisted in unraveling the mechanisms and signaling pathways of auxin-regulated developmental programs (Fukui and Hayashi, 2018; Ma et al., 2018).

Auxin is perceived in the nucleus via the TRANSPORT INHIBITOR RESISTANT1 (TIR1)/AUXIN SIGNALING F-BOX (AFB)-AUXIN/INDOLE-3-ACETIC ACID (AUX/IAA) co-receptor complex within the S-PHASE KINASE-ASSOCIATED PROTEIN1-CULLIN1-F-BOX (SCF) E3 ligase (Calderon-Villalobos et al., 2010). The formation of the SCF^TIR1/AFB^–AUX/IAA–auxin complex promotes the degradation of the AUX/IAA transcriptional repressors, releasing AUXIN RESPONSE FACTOR (ARF) transcription factors from repression, which in turn leads to an auxin-induced transcriptional response (Weijers and Wagner, 2016). Recently, we isolated and characterized a set of small, synthetic molecules affecting plant development, called RubNeddins (RNs), to which mutants deficient in the enzyme subunit AUXIN RESISTANT1 (AXR1) are less sensitive (Vain et al., 2019). AXR1 activates RELATED TO UBIQUITIN/NEURAL PRECURSOR CELL EXPRESSED DEVELOPMENTALLY DOWNREGULATED PROTEIN8 (RUB/NEDD8), which in turn regulates the SCF complex by modifying CULLIN1 (Calderon-Villalobos et al., 2010). The RN molecules differentially affect the interactions of TIR1 with specific AUX/IAAs, dependent on the experimental conditions, leading to regulation of selective plant developmental aspects (Vain et al., 2019). One of these molecules, RN 1 (RN1), strongly promotes hypocotyl elongation in Arabidopsis (*Arabidopsis thaliana*) seedlings at concentrations which only weakly inhibit root elongation (Vain et al., 2019).

In this study, we took advantage of these selective effects of RN1 to dissect the genetic regulation of hypocotyl elongation in Arabidopsis. Hypocotyl elongation following seed germination occurs mainly via cell expansion and is regulated by multiple internal and environmental signals including hormone signaling, circadian rhythm, gravity, and light (Gendreau et al., 1997; Vandenbussche et al., 2005). Auxin in particular has long been known to play an important role in hypocotyl elongation in light conditions (Gray et al., 1998; Jensen et al., 1998). We confirmed RN1 promotes light-grown hypocotyl elongation via the SCF^TIR1/AFB^ complex. Our results suggest RN1 treatment triggers an auxin perception module including the receptors TIR1, AFB2, and/or AFB5, leading to degradation of distinct AUX/IAAs, such as IAA-INDUCIBLE7 (IAA7). Using a transcriptome profiling strategy, we identified three stress-related genes, namely *ZINC FINGER OF ARABIDOPSIS THALIANA10* (*ZAT10*), *ARABIDOPSIS TOXICOS EN LEVADURA31* (*ATL31*), and *WRKY DNA-BINDING PROTEIN33* (*WRKY33*), which are rapidly upregulated by RN1 treatment, despite being downregulated by treatment with the synthetic auxin 2,4-dichlorophenoxyacetic acid (2,4-D). Our results suggest these three genes take part in a common regulatory network downstream of a distinct auxin perception module, which is involved in the selective promotion of hypocotyl elongation.

## Results

### RN1-induced hypocotyl elongation requires the SCF^TIR1/AFB^ complex

**Figure 1:**
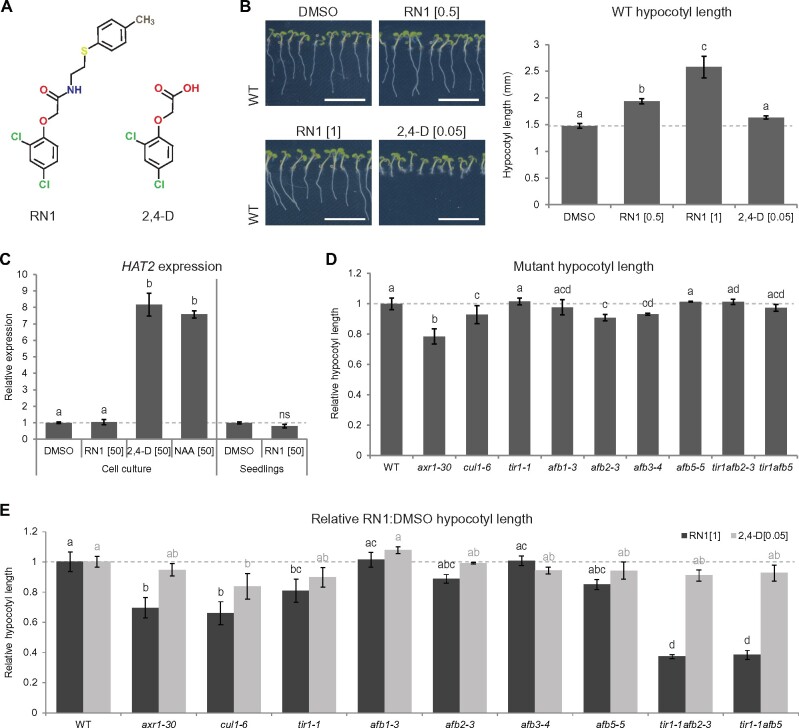
RN1 effects differ from those of direct 2,4-D treatment and RN1 induces hypocotyl elongation through the SCF^TIR1/AFB^ pathway. A, Chemical structure of RN1 and 2,4-D. B, Seedling phenotype (left) and hypocotyl length (right) in 5-d-old wild-type (WT) seedlings treated with RN1 or 2,4-D for 5 d. Representative images are shown. Scale bars represent 1 cm. C, Expression of *HAT2* relative to DMSO treatment after 30 min of RN1, 2,4-D, and NAA treatment in cell cultures and after 30 min of RN1 treatment in 5-d-old WT seedlings. D, E, Hypocotyl length relative to the WT (D) and relative to DMSO treatment after 5 d of RN1 and 2,4-D treatments, relative to that in WT (E) in 5-d-old *axr1-30*, *cul1-6*, *tir1-1*, *afb1-3*, *afb2-3*, *afb3-4*, *afb5-5*, *tir1-1afb2-3*, and *tir1-1afb5* mutants. For all graphs, means of at least three biological replicates are shown, horizontal dashed lines represent DMSO (B, C) or WT (D, E) and error bars represent se of the mean. Different letters (of the same color) indicate statistical differences according to the Tukey–Kramer honestly significant difference test or Wilcoxon rank-sum test at α  =  0.05 (B–E); ns indicates no significant difference compared to DMSO according to the Student’s *t* test at α  =  0.05 (C); letters of different colors (black and gray) indicate two different groups of samples (RN1 and 2,4-D treatments) that were not statistically compared (E). Concentrations in square brackets are in µM.

RN1 is a synthetic molecule (Figure 1A), identified from an AXR1-dependent developmental plant-based chemical screen as selectively stimulating hypocotyl elongation and inhibiting primary root growth in seedlings of Arabidopsis (Vain et al., 2019). We showed previously that the growth of seedlings on RN1-supplemented medium strongly induced hypocotyl elongation in a dose-dependent manner up to a concentration of 2 µM and inhibited primary root growth at concentrations above 0.5 µM (Vain et al., 2019). The negative effect of RN1 on primary root growth was weak at concentrations of 0.5–1 µM, at which the positive effect on hypocotyl length was rather strong. We confirmed RN1 enhances Arabidopsis hypocotyl elongation at these two concentrations, with 1 µM having a stronger effect (Figure 1B).

Structurally, part of the RN1 molecule closely resembles the synthetic auxin 2,4-D (Figure 1A) and it has previously been shown RN1 can be slightly metabolized to release some 2,4-D in planta (Vain et al., 2019). We found 2,4-D may slightly increase hypocotyl length at a treatment concentration of 0.05 µM (Figure 1B). We therefore further investigated the effects of 2,4-D at a range of concentrations by performing a dose response analysis, revealing a weak activity of 2,4-D on promotion of hypocotyl elongation (Supplemental Figure S1A) at concentrations that strongly inhibited primary root growth (Supplemental Figure S1B). These results show the effects of RN1 and 2,4-D treatment on hypocotyl elongation seem to be quite different, suggesting one of several possible alternatives. It may be the activity of RN1 on the promotion of hypocotyl elongation is independent of its structural analogy to 2,4-D. Alternatively, RN1 may act as a bipartite prohormone as has been demonstrated for other 2,4-D analogs (Savaldi-Goldstein et al., 2008), enabling easier uptake than 2,4-D itself into hypocotyl tissue and/or providing a means for low-level, chronic 2,4-D release in planta, resulting in different effects than those of direct, acute treatment with the potent 2,4-D molecule. The potential for prohormone activity of RN1 is strengthened by the observations that RN1-enhanced hypocotyl elongation is almost abolished by removal or slight alteration of the 2,4-D substructure (Vain et al., 2019).

Auxin is known to be a major regulator of plant growth and hypocotyl development (Spartz et al., 2014). Therefore, we tested the effects of short-term RN1 treatments on expression of the auxin early-responsive homeobox gene *HOMEOBOX FROM ARABIDOPSIS THALIANA2* (*HAT2*; Sawa et al., 2002). We reasoned the use of rather high treatment concentrations would ensure early-responsive genes could be easily identified in spite of the short treatment time. While 30 min treatments with 50-µM 2,4-D and another synthetic auxin, 1-naphthaleneacetic acid (NAA), strongly promoted *HAT2* expression in Arabidopsis cell suspension cultures, RN1 did not affect the level of *HAT2* expression, neither in cell cultures nor in seedlings (Figure 1C). Overall, our results show the synthetic molecule RN1, despite its 2,4-D-like substructure, induces distinct auxin-like effects that differ to those of direct 2,4-D treatment. With this in mind, the selective effects of RN1 on hypocotyl elongation prompted us to take advantage of RN1 as a tool to investigate hypocotyl regulation in a way not possible using 2,4-D itself.

RN1 requires the SCF^TIR1/AFB^ complex to induce hypocotyl elongation (Vain et al., 2019). Interestingly, we found Arabidopsis single mutants deficient in the RUB/NEDD8-activating enzyme subunit AXR1 (*axr1-30*), in the SCF complex component CULLIN1 (*cul1-6*), or in the F-box proteins AFB2 or 3 (*afb2-3* and *afb3-4*) displayed shorter hypocotyls compared to the wild-type (Figure 1D). This suggests a role of auxin signaling mediated by the SCF complex and in particular the receptors AFB2 and AFB3 in hypocotyl elongation. As was previously shown for the mutants *axr1-30*, *cul1-6*, *tir1-1*, and *tir1-1afb1-3afb3-4* (Vain et al., 2019), we showed the mutants *axr1-30*, *cul1-1*, and *tir1-1* were significantly less sensitive, while *afb2-3* and *afb5-*5 were slightly less sensitive, than the wild-type to the effects of 1-µM RN1 on hypocotyl elongation (Figure 1E). Furthermore, the potency of RN1 on hypocotyl elongation was more strongly reduced in the double mutants *tir1-1afb2-3* and *tir1-1afb5* compared to the wild-type, than in the relevant single mutants (Figure 1E), suggesting redundancy between TIR1 and AFB2/AFB5. Interestingly, *cul1-6* was the only mutant that displayed significantly reduced sensitivity to 2,4-D than the wild-type, supporting the better potential of RN1 as a tool to dissect hypocotyl growth regulation via TIR1/AFB signaling pathways. Together, these data demonstrate that RN1-mediated hypocotyl elongation requires the SCF^TIR1/AFB^ complex and in particular the auxin receptors TIR1, AFB2, and AFB5.

### Direct RN1 and 2,4-D treatments induce different early transcriptomic responses

To identify molecular actors involved in hypocotyl elongation, an RN1-dependent transcriptomic analysis was performed. First, a pilot cDNA-amplified fragment length polymorphism (AFLP) experiment was conducted on 7-d-old Arabidopsis seedlings to determine the optimum conditions for full transcriptomic profiling. We focused on early transcriptional modulation using treatments of 30 min and 3 h of 1-µM RN1. Interestingly, the transcriptional response differed considerably between these two durations of RN1 treatment and suggested the occurrence of an early transcriptional response to RN1. The expression levels of 13 genes were modulated after 30 min of RN1 treatment, while only one gene expression was changed after 3 h, with no overlap in the gene identity (Supplemental Table S1). Among the thirteen genes regulated after 30-min RN1 treatment, eleven were induced and only two were repressed (Supplemental Table S1). Therefore, the cDNA-AFLP analysis revealed RN1 induces an early transcriptomic response, within 30 min, at a micromolar concentration range.

**Figure 2:**
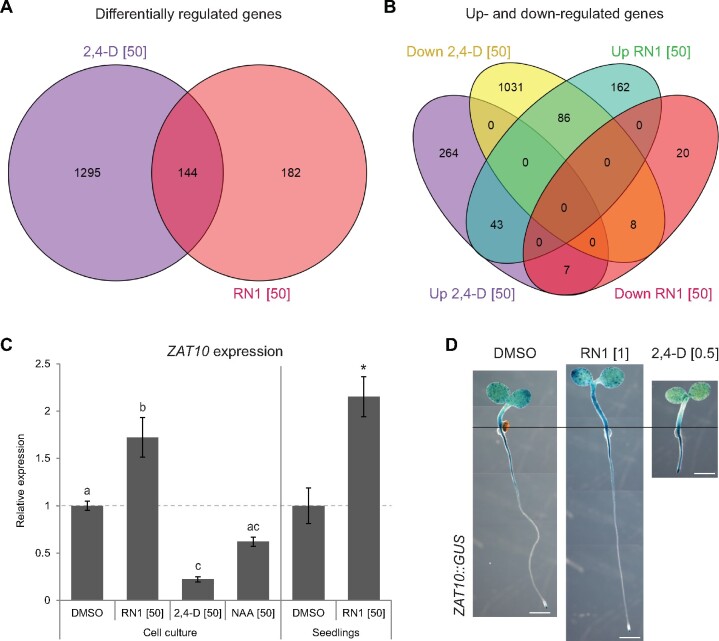
Differentially expressed genes differ after direct RN1 and 2,4-D treatments. A, B, Venn diagrams of the numbers of genes significantly differentially regulated (A) or specifically up- and downregulated (B) by RN1 or 2,4-D treatment of 30 min in cell culture as determined by RNAseq. Genes with expression log2 fold change ≥0.5 after 2,4-D or RN1 treatment compared to DMSO treatment, with a *P*-value cutoff corrected for the false discovery rate set at 0.05, were considered as significantly differentially regulated. C, Expression of *ZAT10* relative to DMSO treatment after 30 min of RN1, 2,4-D, and NAA treatment in cell cultures and after 30 min of RN1 treatment in 5-d-old WT seedlings. Means of at least three biological replicates are shown, horizontal dashed line represents WT DMSO and error bars represent se of the mean. Different letters indicate statistical differences according to the Tukey–Kramer honestly significant difference test at α  =  0.05; asterisk indicates significant difference compared to DMSO according to the Student’s *t* test at α  =  0.05. D, Activity of RN1 and 2,4-D on *ZAT10* expression in *ZAT10::GUS* seedlings treated with RN1 or 2,4-D for 5 d. Whole-plant images are composed of multiple images. Scale bars represent 1 mm. Horizontal black line marks root-hypocotyl junctions. Concentrations in square brackets are in µM.

Based on these results, an RNA sequencing (RNAseq) experiment was conducted on samples treated for 30 min with RN1. To compare the transcriptomic responses induced by direct RN1 and 2,4-D treatments, samples treated for 30 min with 2,4-D were also included. With the aim of more easily distinguishing early-responsive genes, we again chose a high treatment concentration of 50 µM for both molecules. To ensure a homogeneous response, the RNAseq analysis was performed using Arabidopsis cell suspension cultures, as was previously used for profiling methyl jasmonate (Pauwels et al., 2008). The expression of a total of 326 genes was significantly affected by the RN1 treatment, while the expression of 1,439 genes was modulated by the 2,4-D treatment, compared to the control treatment (Figure 2A;  Supplemental Table S2, A and B). These results suggest RN1 is less potent than 2,4-D in altering gene expression after a 30-min direct treatment and might therefore induce a more specific early transcriptomic response. Of the genes affected by either of these molecules, 144 (8.9%) were commonly regulated by both molecules (Figure 2A;  Supplemental Table S2C). The 2,4-D treatment mainly suppressed gene expression, with 1,125 (78.2%) of the 2,4-D-modulated genes being downregulated and 314 (21.8%) being upregulated (Figure 2B;  Supplemental Table S2B). In contrast, RN1 mainly induced gene expression, as 291 (89.3%) of the RN1-modulated genes were upregulated and only 35 (10.7%) were downregulated (Figure 2B;  Supplemental Table S2A). Additionally, 93 (64.6%) of the 144 genes regulated by both molecules displayed an opposite pattern of regulation, with gene expression being induced by one molecule and repressed by the other (Figure 2B;  Supplemental Table S2D). Of the 248 genes specifically induced by RN1, 86 (34.7%) were downregulated by 2,4-D (Figure 2B;  Supplemental Table S2D) and we chose to focus on this gene set, with the aim of isolating genes specifically involved in RN1-mediated hypocotyl elongation.

As the RNAseq was performed in cell cultures, we compared the effects of RN1 on gene expression in cell cultures and seedlings. We used reverse transcription quantitative real-time polymerase chain reaction (RT-qPCR) to investigate the expression of *ZAT10*, which was identified as an RN1-upregulated gene in both the cDNA-AFLP and RNAseq experiments (Supplemental Tables S1 and S2A). In agreement with the RNAseq data, while 30-min treatment of cell cultures or seedlings with 50-µM RN1 significantly enhanced *ZAT10* expression, 30-min treatment with 50-µM 2,4-D significantly repressed the *ZAT10* expression (Figure 2C). Additionally, 30-min treatment with 50-µM NAA slightly, but not significantly, decreased the *ZAT10* expression (Figure 2C). To investigate the effects of RN1 on the expression pattern of *ZAT10*, we performed GUS staining on seedlings of *ZAT10::GUS* grown for 5 d on chemical-supplemented medium. The results showed *ZAT10* is expressed mainly in the cotyledons, lower hypocotyl, upper root, and root tip in control conditions (Figure 2D). The *ZAT10* expression was noticeably increased in the hypocotyl, especially the upper hypocotyl, in response to 1-µM RN1 treatment, but not in response to 0.5-µM 2,4-D treatment, which rather increased the *ZAT10* expression in the root tip (Figure 2D). These results imply both the level and pattern of the *ZAT10* expression are affected differently by direct treatments with RN1 and 2,4-D as well as suggesting the possibility *ZAT10* may be involved in the effect of RN1 on hypocotyl elongation. In conclusion, the RNAseq analysis suggests RN1 acts mainly as a promotor of gene expression, with many RN1-induced genes being subject to downregulation by direct 2,4-D treatment. These results reveal a set of genes, including *ZAT10*, with the potential for being involved in RN1-mediated hypocotyl elongation.

### The early transcriptomic response to RN1 requires the SCF^TIR1/AFB^ signaling pathway

**Figure 3:**
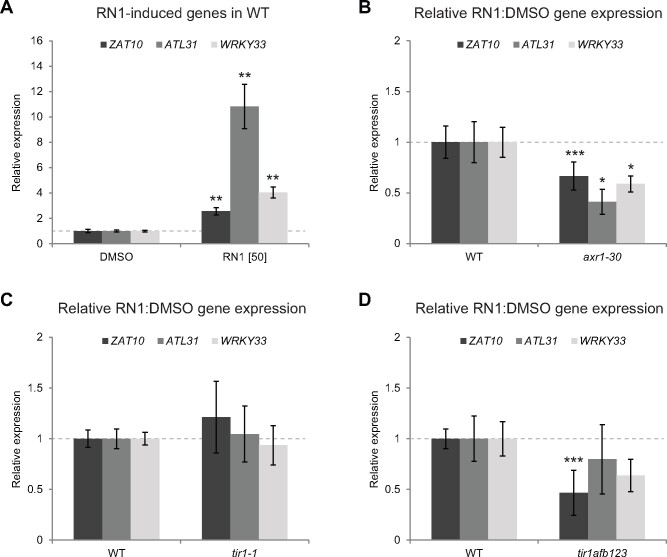
RN1 requires the SCF^TIR1/AFB^ complex to promote gene expression. A, Expression of *ZAT10*, *ATL31*, and *WRKY33* relative to DMSO treatment after 30 min of RN1 treatment in 5-d-old wild-type (WT) seedlings. Concentrations in square brackets are in µM. B–D, Expression of *ZAT10*, *ATL31I*, and *WRKY33* relative to DMSO treatment after 30 min of 50-µM RN1 treatment in 5-d-old *axr1-30* (B), *tir1-1* (C), and *tir1-1afb1-3afb2-3afb3-4* (*tir1afb123*) (D) mutants, relative to that in WT. Means of at least three biological replicates are shown, horizontal gray lines represent DMSO (A) or WT (B–D) and error bars represent SE of the mean. Dots/asterisks indicate significant differences compared to DMSO (A) or WT (B–D) with *P *<* *0.05 (*), *P *<* *0.01 (**), or *P* < 0.1 (***) according to the Student’s *t* test or Wilcoxon rank-sum test.

To understand the specificity of the RN1 transcriptional response and its effect on hypocotyl elongation, we focused on *ZAT10* and two other candidate genes selected from the same gene set (upregulated by RN1 treatment and downregulated by 2,4-D treatment; Supplemental Table S2D), which are all known to be involved in stress responses. *ZAT10* has been implicated in several abiotic stress responses (Sakamoto et al., 2000, 2004; Mittler et al., 2006; Rossel et al., 2007). The other candidates selected were *ATL31* and *WRKY33*, which have both been implicated in defense responses to pathogens (Zheng et al., 2006; Maekawa et al., 2012), while *ATL31* is additionally involved in carbon and nitrogen balance responses (Sato et al., 2009; Maekawa et al., 2012). Furthermore, evidence has suggested crosstalk between *ATL31* and *WRKY33* (Maekawa et al., 2012; Reyes et al., 2015). To confirm the RNAseq data obtained with cell cultures, we performed RT-qPCR analysis of these genes in 5-d-old Arabidopsis seedlings after 30 min of RN1 treatment at 50 µM. The results revealed RN1 also induces the expression of these selected candidates in seedlings (Figure 3A).

The SCF^TIR1/AFB^ complex requirement for hypocotyl elongation induction by RN1 (Figure 1E) prompted us to study the expression of the candidate genes in AXR1- and TIR1/AFB-deficient Arabidopsis mutants after RN1 treatment. The mutant *axr1-30* displayed significantly less sensitivity to RN1-induced expression of the three candidate genes compared to the wild-type (Figure 3B). This suggests RN1 induction of these genes requires the AXR1 signaling pathway. Although the mutant *tir1-1* was slightly less sensitive to RN1 compared to the wild-type in terms of hypocotyl elongation (Figure 1E), the RN1-induced expression of *ZAT10*, *ATL31*, and *WRKY33* in *tir1-1* and the wild-type were similar (Figure 3C), which might be explained by redundancy of TIR1 with the other members of the TIR1/AFB protein family. The gene expression data were much less robust than the hypocotyl elongation data, displaying far more variation across biological replicates; therefore, we reasoned higher-order multiple *tir1*/*afb* mutations may be required to confer resistance to RN1-induced changes in gene expression. Thus, we next analyzed the expression levels of the candidate genes in the quadruple TIR1/AFB-deficient mutant *tir1-1afb1-3afb2-3afb3-4*, revealing a trend of lowered sensitivity to RN1-induced expression of the candidate genes; however, only *ZAT10* expression was significantly less sensitive to RN1 in this mutant (Figure 3D). These results suggest RN1 induction of *ZAT10*, *ATL31*, and *WRKY33* occurs through the SCF^TIR1/AFB^ auxin signaling pathway.

Overall, these data imply the involvement of AXR1 and the SCF^TIR1/AFB^ complex in the RN1-induced early transcriptional response and furthermore suggest the possibility the candidate genes *ZAT10*, *ATL31*, and/or *WRKY33* may be involved in the RN1 mode of action on hypocotyl elongation.

### Gain of IAA7 function results in loss of RN1 effects

**Figure 4:**
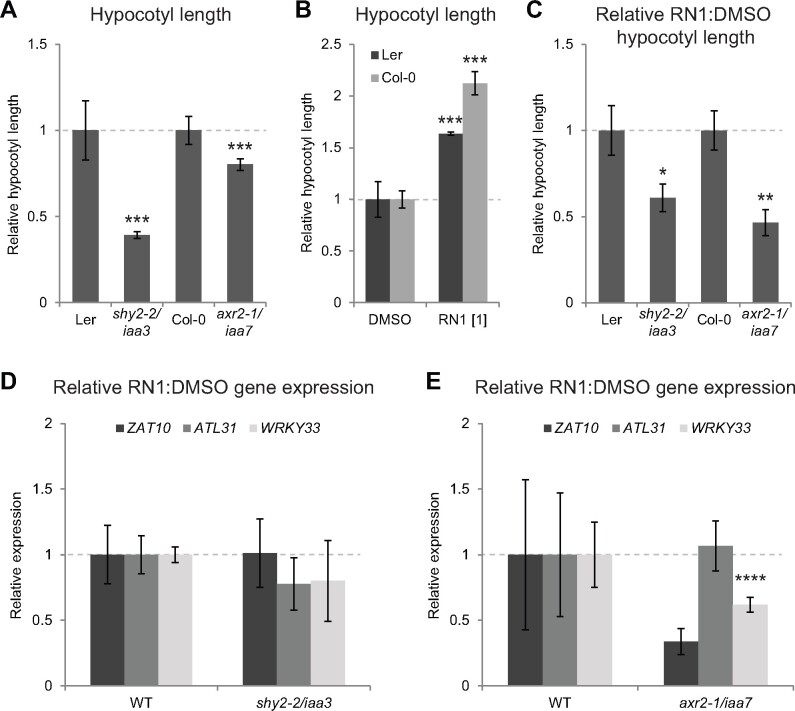
RN1-induced hypocotyl elongation and gene expression likely occur downstream of IAA7 degradation. A, Hypocotyl length relative to the wild-type (WT; Ler and Col-0, respectively) in 5-d-old AUX/IAA gain-of-function mutants *shy2-2*/*iaa3* and *axr2-1/iaa7*. B, Hypocotyl length relative to DMSO treatment in 5-d-old WT (Ler and Col-0) seedlings after 5 d of RN1 treatment. Concentrations in square brackets are in µM. C, Hypocotyl length relative to DMSO treatment after 5 d of 1-µM RN1 treatment in 5-d-old *shy2-2*/*iaa3* and *axr2-1*/*iaa7* mutants, relative to that in WT (Ler and Col-0, respectively). D–E, Expression of *ZAT10*, *ATL31*, and *WRKY33* relative to DMSO treatment after 30 min of 50-µM RN1 treatment in 5-d-old *shy2-2*/*iaa3* (D) and *axr2-1*/*iaa7* (E) mutants, relative to that in WT (Ler and Col-0, respectively). Means of at least three biological replicates are shown, horizontal dashed lines represent WT (A, C–E) or DMSO (B) and error bars represent se of the mean. Dots/asterisks indicate significant differences compared to the relevant WT (A, C), DMSO for the same line (B) or WT for the same gene (D–E) with , *P < 0.05, **P < 0.01, ***P < 0.001, or ****P < 0.1 according to the Student’s *t* test or Wilcoxon rank-sum test.

The transcriptional repressors IAA3 and IAA7 have been implicated in regulating hypocotyl elongation (Timpte et al., 1992; Reed et al., 1998) and we previously showed RN1 selectively promotes the degradation of these two AUX/IAAs in planta (Vain et al., 2019). We therefore tested the sensitivity of the AUX/IAA gain-of-function mutants *shy2-2/iaa3* and *axr2-1/iaa7* to RN1 as a promoter of hypocotyl elongation. First, we confirmed the hypocotyls of these two mutants were shorter than the wild-type in control conditions (Figure 4A), as has been shown previously (Timpte et al., 1992; Reed et al., 1998). Interestingly, in agreement with our previous work showing variable RN sensitivity between different Arabidopsis accessions (Vain et al., 2019), *Landsberg erecta* (Ler) and Columbia-0 (Col-0) showed different sensitivities to RN1-induced hypocotyl elongation, with Ler being substantially less sensitive than Col-0 (Figure 4B). We found both *shy2-2*/*iaa3* (Ler background) and *axr2-1*/*iaa7* (Col-0 background) were significantly less sensitive than their respective wild-types to RN1-induced hypocotyl elongation (Figure 4C), suggesting the degradation of IAA3 and IAA7 may be important for this effect of RN1. We next investigated the sensitivities of these gain-of-function mutants to RN1-induced upregulation of our three candidate genes. While *shy2-2*/*iaa3* showed similar sensitivity to Ler in terms of RN1-induced *ZAT10*, *ATL31*, and *WRKY33* expression (Figure 4D), *axr2-1*/*iaa7* showed less sensitivity than Col-0 in terms of RN1-induced *ZAT10* and *WRKY33* expression, but similar sensitivity to Col-0 in terms of RN1-induced *ATL31* expression (Figure 4E). Together, these results suggest RN1 promotion of hypocotyl elongation and at least part of the RN1-induced early transcriptional response occur downstream of the degradation of specific AUX/IAAs, most likely including IAA7.

### *ZAT10*, *ATL31*, and *WRKY33* act in a common gene regulatory network

**Figure 5:**
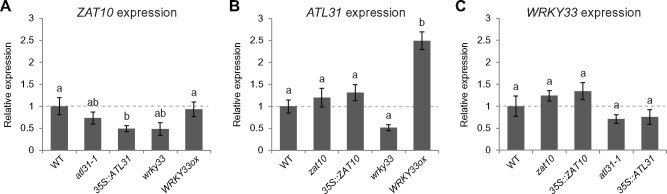
*ZAT10*, *ATL31*, and *WRKY33* act in a common regulatory gene expression network. A–C, Expression of *ZAT10* (A), *ATL31* (B), and *WRKY33* (C) relative to the WT in 5-d-old mutant and overexpressor lines of *ZAT10* (*zat10* and *35S::ZAT10*), *ATL31* (*atl31-1* and *35S::ATL31*), and *WRKY33* (*wrky33* and *WRKY33ox*). Means of at least three biological replicates are shown, horizontal dashed lines represent WT and error bars represent se of the mean. Different letters indicate statistical differences according to the Tukey–Kramer honestly significant difference test or Wilcoxon rank-sum test at α = 0.05.

To determine whether *ZAT10*, *ATL31*, and *WRKY33* may belong to the same transcriptional network, potentially regulating hypocotyl elongation triggered by RN1, their gene transcript levels were analyzed in mutant and overexpressor Arabidopsis lines of these genes. Interestingly, *ZAT10* expression was significantly reduced in the *ATL31* overexpressor line as well as being noticeably, but not significantly, reduced in the *wrky33* mutant (Figure 5A), suggesting *ATL31* and *WRKY33* may negatively and positively regulate *ZAT10* expression, respectively. *ATL31* expression was noticeably, but not significantly, reduced in the *wrky33* mutant and was significantly enhanced in the *WRKY33* overexpressor line (Figure 5B), implying *WRKY33* acts as a positive regulator of *ATL31* expression. *WRKY33* expression was not significantly modulated in any of the lines (Figure 5C), suggesting *WRKY33* may act upstream of the other genes.

To investigate the possibility that these genes interact in an RN1-induced regulatory network, we tested the effects of RN1 on their expression in mutant and overexpressor lines of the genes. We found *ZAT10* expression was significantly more enhanced by RN1 treatment in the *ATL31* overexpressor line than in the wild-type (Supplemental Figure S2A). Additionally, *ATL31* expression was significantly less sensitive to RN1 in the *WRKY33* overexpressor line than in the wild-type (Supplemental Figure S2B), most likely because *ATL31* expression is already significantly increased in the *WRKY33* overexpressor line without treatment (Figure 5B). Although *WRKY33* expression tended to be more sensitive to RN1 in *ATL31*-affected lines than in the wild-type, the differences were not significant (Supplemental Figure S2C).

Interestingly, both *ZAT10* and *WRKY33* showed the trend of higher sensitivity to RN1-induced expression than the wild-type in the *atl31-1* mutant and even more so in the *ATL31* overexpressor line (Supplemental Figure S2, A and C), suggesting a balanced expression of *ATL31* may be important for regulating the responses of the other two genes to RN1. Furthermore, *ZAT10* and *ATL31* both showed the trend of lower sensitivity to RN1 than the wild-type in the *WRKY33* overexpressor line (Supplemental Figure S2, A and B). Paradoxically, although *WRKY33* appears to act upstream of the two other genes, enhancing their expression in the absence of RN1, RN1 does not require *WRKY33* to enhance the expression of the other two genes and their RN1-enhanced expression appears to be suppressed by *WRKY33*. One potential explanation for these results is the functional redundancy known to occur within the large family of WRKY transcription factors (Eulgem and Somssich, 2007). Together, these results suggest these three genes may be involved in a regulatory interaction network stimulated by RN1 treatment, with *WRKY33* likely acting upstream as a positive regulator of *ATL31* and *ZAT10* expression, and *ATL31* acting as a suppressor of *ZAT10* expression.

### RN1-induced genes are potential actors in hypocotyl elongation

To investigate whether the induction of the *ZAT10*, *ATL31*, and *WRKY33* regulatory network by RN1 may be involved in the RN1 promotion of hypocotyl growth, we next investigated the mutant and overexpressor lines for their hypocotyl growth and their sensitivity to RN1-induced hypocotyl elongation. To further investigate the potential interactions between these three genes in the regulation of hypocotyl elongation, we also included homozygous double mutant combinations of the genes in our analyses.

**Figure 6:**
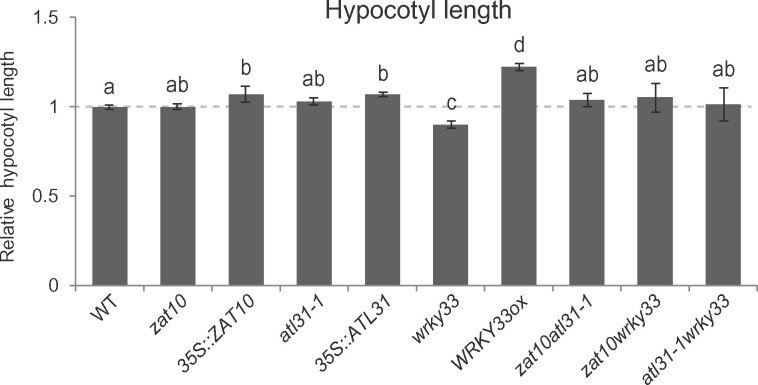
Hypocotyl elongation is altered in lines affected in *ATL31* and *WRKY33*. Hypocotyl length relative to the WT in 5-d-old mutant and overexpressor lines of *ZAT10*, *ATL31*, and *WRKY33*. Hypocotyl length was analyzed in mutants and overexpressor lines of *ZAT10* (*zat10* and *35S::ZAT10*), *ATL31* (*atl31-1* and *35S::ATL31*), and *WRKY33* (*wrky33* and *WRKY33ox*) as well as in the homozygous double mutants *zat10atl31-1*, *zat10wrky33*, and *atl31-1wrky33*. Means of at least three biological replicates are shown, horizontal gray line represents WT and error bars represent se of the mean. Different letters indicate statistical differences according to the Tukey–Kramer honestly significant difference test at α = 0.05.

In control conditions, the overexpressor lines of *ZAT10*, *ATL31*, and *WRKY33* all had significantly longer hypocotyls, while the *wrky33* mutant had significantly shorter hypocotyls, than the wild-type (Figure 6). These results support roles for *ZAT10*, *ATL31*, and *WRKY33* in hypocotyl growth. Furthermore, the *atl31-1* mutant was significantly more sensitive, while the *WRKY33* overexpressor line was significantly less sensitive, to RN1-induced hypocotyl elongation than the wild-type (Supplemental Figure S3). These findings imply *ATL31* and *WRKY33* are specifically involved in RN1-induced hypocotyl elongation.

Interestingly, combination of the *wrky33* mutation with either the *zat10* or *atl31-1* mutation rescued the short hypocotyl phenotype of single *wrky33* mutant in control conditions to that of the wild-type (Figure 6). Moreover, combination of the *atl31-1* mutation with either the *zat10* or *wrky33* mutation rescued the increased sensitivity of the *atl31-1* single mutant to RN1-enhanced hypocotyl length to that of the wild-type (Supplemental Figure S3). These results strengthen the hypothesis *ZAT10*, *ATL31*, and *WRKY33* are inter-dependent genes acting in the same regulatory transcriptional network.

Together, the results of this study suggest hypocotyl elongation in light-grown Arabidopsis seedlings is regulated by a gene network involving *ZAT10*, *ATL31*, and *WRKY33*, acting downstream of a specific auxin perception module likely involving the SCF^TIR1/AFB^ complex F-boxes TIR1, AFB2, and AFB5 and the AUX/IAA transcriptional repressor IAA7. We postulate RN1 stimulates this signaling pathway by selectively promoting the interaction of these F-box receptors with the transcriptional repressor IAA7, leading to IAA7 degradation and the consequent activation of downstream signaling, which in turn leads to upregulation of *WRKY33* expression (Supplemental Figure S4). *WRKY33* acts upstream of *ATL31* and *ZAT10*, in turn promoting their expression, while *ATL31* acts as an inhibitor of *ZAT10* expression, with this interactive gene network contributing to the regulation of hypocotyl elongation (Supplemental Figure S4).

## Discussion

Auxin is involved in regulating virtually all aspects of plant development, its diverse effects being mediated through the degradation of a multitude of AUX/IAA transcriptional repressors. The AUX/IAAs, of which there are 29 in Arabidopsis, vary considerably in their auxin-induced degradation rates and the specificity of their interactions with the six Arabidopsis TIR1/AFB F-box proteins, thus allowing a variety of complex auxin responses, specific to certain aspects of plant growth and development (Weijers and Wagner, 2016). We showed in the present work that mutants deficient in TIR1, AFB2, and AFB5 are resistant to RN1-induced hypocotyl elongation, implying these particular F-box receptors may be involved in a selective auxin-sensing module regulating hypocotyl growth. Various *tir1*, *afb1*, *afb2*, and *afb3* mutant combinations have previously been shown to be resistant to IAA-induced hypocotyl elongation (Chapman et al., 2012), while the *afb5* mutant is highly resistant to hypocotyl elongation induced by the synthetic auxin picloram (Greenham et al., 2011). Our results add further support for important roles of selective SCF^TIR1/AFB^ signaling in auxin-induced hypocotyl elongation.

Hypocotyl elongation has been shown to be defective, in both light and dark conditions, in *shy2* and *axr2* mutants (Timpte et al., 1992; Reed et al., 1998), which were later revealed as gain-of-function mutants of the AUX/IAAs IAA3 and IAA7, respectively (Tian and Reed, 1999; Nagpal et al., 2000). Furthermore, the IAA7 loss-of-function mutant *axr2-5* was shown to display slightly longer light-grown hypocotyls than the wild-type (Nagpal et al., 2000). Therefore, auxin may induce hypocotyl elongation via the selective degradation of IAA3 and/or IAA7, by releasing the repression of their interacting auxin response-activating ARFs. In agreement with this hypothesis, we previously showed RN1, which strongly induces hypocotyl elongation, selectively promotes the degradation of IAA3 and IAA7 in planta (Vain et al., 2019). In the present study, we showed *shy2-2*/*iaa3* and *axr2-1*/*iaa7* mutants are resistant to RN1-induced hypocotyl elongation, thus supporting an important role for auxin-induced degradation of IAA3 and IAA7 in regulating hypocotyl growth. The mutant *axr2-1/iaa7* is additionally resistant to RN1-induced expression of *ZAT10* and *WRKY33*, which suggests the RN1-induced gene network regulating hypocotyl elongation may be upregulated downstream of IAA7 degradation. The *axr2-1/iaa7* mutant was previously shown to be resistant to hypocotyl elongation induced by IAA and the synthetic auxin picloram (Chapman et al., 2012), therefore our results add further support for an important role of IAA7 degradation in auxin-induced hypocotyl elongation. Additionally, *axr5-1*/*iaa1* and *slr-1*/*iaa14*, but not *msg2-1*/*iaa19*, showed resistance to IAA- and picloram-induced hypocotyl elongation (Chapman et al., 2012), suggesting roles for other selective AUX/IAAs in this process as well.

Interestingly, RN1 also induces adventitious root formation (Vain et al., 2019), a developmental program recently shown to be regulated by selective auxin co-receptor complexes also specifically involving TIR1 and AFB2 and in this case the AUX/IAAs IAA6, IAA9, and/or IAA17 (Lakehal et al., 2019). RN1, therefore, presents an interesting potential additional application in investigations of adventitious rooting regulatory mechanisms.

ARF6, ARF7, and ARF8 have been implicated in hypocotyl elongation. The genes encoding these three ARFs are expressed in the hypocotyl (Chapman et al., 2012), and in untreated conditions, null *arf6*, *arf7*, and *arf8* mutants display shorter dark-grown hypocotyls than the wild-type (Reed et al., 2018). Dark-grown hypocotyls are shorter in double mutants of these ARFs than the single mutants and even shorter in a triple mutated/silenced line, being similar in length to those of *shy2* and *axr2* seedlings (Reed et al., 2018). These results suggest IAA3 and IAA7 as well as ARF6, ARF7, and ARF8 are important for etiolation of hypocotyls in the dark. In the case of light-grown hypocotyl growth, treatment with the synthetic auxin-like compound 533, which strongly promotes hypocotyl elongation in the wild-type (Savaldi-Goldstein et al., 2008), is less effective on the mutants *shy2* and *axr2* and the triple mutated/silenced line affected in ARF6, ARF7, and ARF8 (Reed et al., 2018). These results strongly support the involvement of IAA3 and IAA7, as well as suggesting the importance of ARF6, ARF7, and ARF8, in hypocotyl elongation induced by auxin. These ARFs, once released from IAA3- and IAA7-mediated repression, are likely to activate further genes and gene networks that lead to hypocotyl cell elongation including, for example, genes with cell wall-loosening functions.

Our comparison of early RN1 and 2,4-D-regulated genes revealed rather little overlap between the two treatments in terms of commonly up- or downregulated genes. Interestingly, comparison of our transcriptomic data with that of a previous microarray analysis of hypocotyls in response to 30 min of picloram treatment (Chapman et al., 2012) reveals a small group of upregulated genes in common between 2,4-D and picloram that are not affected by RN1. This particular group includes several known auxin-related genes such as *GRETCHEN HAGEN3.2* (*GH3.2*), *AUXIN UP-REGULATED F-BOX PROTEIN1* (*AUF1*), and the AUX/IAA *AUXIN-INDUCIBLE2-*27*/IAA5* (*AUX2-27*/*IAA5*). Moreover, only one gene, the AUX/IAA *AXR5/IAA1*, was commonly upregulated by all three treatments. This comparison suggests RN1 may induce a more specific early transcriptomic response than synthetic auxins such as 2,4-D or picloram. This idea is further supported by our finding that RN1 did not affect the expression of *HAT2*, an auxin early-responsive gene, while both 2,4-D and NAA strongly induced its expression.

In this study, we uncovered potential previously unknown roles for genes in hypocotyl elongation, the functions of which may seem surprising. All three of our selected RN1-induced candidate genes, *ZAT10*, *ATL31*, and *WRKY33*, have been shown to play important roles in plant responses to various biotic and abiotic stresses. *ZAT10* was originally identified in a screen for Arabidopsis cDNA clones that confer increased salt resistance in yeast (Lippuner et al., 1996). The encoded protein contains a Cys_2_/His_2_-type zinc finger, a well-characterized DNA-binding motif found in eukaryotic transcription factors (Sakamoto et al., 2000). *ZAT10* expression has been shown to be induced by salt, cold, drought, high light and oxidative, osmotic, and genotoxic stresses, as well as by treatments with the hormones abscisic acid, gibberellin, methyl jasmonate, salicylic acid, and 2,4-D (Sakamoto et al., 2000, 2004; Mittler et al., 2006; Rossel et al., 2007; Pauwels et al., 2008). Furthermore, *ZAT10* overexpressor lines show enhanced resistance to drought, osmotic stress, salinity, heat stress, photoinhibitory light, and exogenous hydrogen peroxide (Sakamoto et al., 2004; Mittler et al., 2006; Rossel et al., 2007), revealing an important role for ZAT10 in plant resistance to a wide variety of abiotic stresses. Interestingly, overexpressor lines of *ZAT10* display growth retardation corresponding with both *ZAT10* expression levels and drought resistance (Sakamoto et al., 2004), highlighting the potential of stress-regulating genes to play simultaneous roles in plant growth.

*ATL* genes, of which there are 80 predicted in Arabidopsis (Serrano et al., 2006), encode a class of E3 ubiquitin ligases containing a Really Interesting New Gene finger, a zinc finger domain that binds to both the corresponding E2 ubiquitin-conjugating enzyme and the substrate (Salinas-Mondragon et al., 1999; Moon et al., 2004). Several of the *ATL* genes, including *ATL2*, *ATL6*, and *ATL31*, are rapidly induced in response to elicitors and pathogen infection (Salinas-Mondragon et al., 1999; Maekawa et al., 2012). Moreover, *ATL6* and *ATL31* overexpression have been shown to enhance resistance to pathogen infection, while *atl31atl6* double mutants show decreased resistance (Maekawa et al., 2012). *ATL31* is, however, perhaps best studied for its role in carbon/nitrogen (C/N) ratio sensing, an important function at the heterotrophic-to-autotrophic seedling transition, at which a growth arrest checkpoint occurs, with high C/N ratios being unfavorable for photosynthesis and therefore resulting in developmental cessation (Sato et al., 2009). The overexpressor lines of *ATL31* are resistant to high C/N stress, while *atl31* mutants are hypersensitive (Sato et al., 2009). 14-3-3 proteins have been revealed as targets for ubiquitination by ATL31, as part of the C/N balance response (Sato et al., 2011). Interestingly, the growth and phenotype effects of C/N stress on *ATL31* overexpressor mutants suggest ATL31 is also important for normal vegetative growth (Sato et al., 2009).

WRKY proteins constitute an important family of transcription factors related to plant defense (Buscaill and Rivas, 2014). Transcriptional reprogramming of the plant in response to pathogen attack involves major changes in gene expression in favor of defense and at the expense of growth and development. There are 74 WRKY members in Arabidopsis, playing both positive and negative roles in defense (Buscaill and Rivas, 2014). Functional redundancy occurs within the WRKY family (Eulgem and Somssich, 2007) and our results suggest potential redundancy of WRKY33 with other WRKY proteins during the response to RN1. WRKY33 is a group I WRKY transcription factor that localizes to the nucleus and contains two DNA-binding domains, which likely bind to cognate W-box cis-elements in the promoters of target genes to regulate their expression (Zheng et al., 2006). While *WRKY33* is generally expressed at low levels in healthy plants, its expression is induced upon infection with pathogenic bacteria and fungi and *wrky33* mutants show enhanced susceptibility to infection by *Botrytis* and *Alternaria brassicicola*, while *35S::WRKY33* plants show enhanced resistance (Zheng et al., 2006). WRKY33 functions therefore in limiting necrotrophic fungal pathogen growth and promoting plant defense, but has also been implicated in tolerance to several abiotic stresses (Zheng et al., 2006; Buscaill and Rivas, 2014). Like other WRKY family members, WRKY33 is phosphorylated by MITOGEN-ACTIVATED PROTEIN KINASEs (MAPKs), but as the phosphorylation does not affect its binding, it is thought rather to promote transactivation activity (Buscaill and Rivas, 2014). WRKY33 forms a complex with MAP KINASE SUBSTRATE1 (MKS1), a substrate of MAPK4, and upon pathogen infection or elicitor treatment, activated MAPK4 phosphorylates MKS1 and releases WRKY33 from the complex (Qiu et al., 2008). The WRKY33-interacting proteins SIGMA FACTOR BINDING PROTEIN1 (SIB1) and SIB2 stimulate the DNA-binding activity of WRKY33 in the nucleus. *SIB1* and *SIB2* are rapidly induced in response to necrotrophic pathogen infection and the mutants *sib1* and *sib2* show enhanced susceptibility to infection while *SIB1*-overexpressing plants show enhanced resistance (Lai et al., 2011).

Interestingly, *WRKY33* has been found to co-express with *ATL31* and *ATL6* and transcript levels of all three genes were highly induced by C/N stress, suggesting potential crosstalk among ATL31, ATL6, and WRKY33 in sensing the C/N balance (Maekawa et al., 2012). Furthermore, WRKY33 has been found to positively regulate *ATL31* expression in Arabidopsis via a specific W-box cis-acting element in the *ATL31* promoter (Reyes et al., 2015). Elicitor-induced *ATL31* expression was lower in the *wrky33* mutant and higher in the *WRKY33* over-expressor line compared to the wild-type, suggesting *ATL31* transcription in response to pathogens lies under the control of WRKY33 (Reyes et al., 2015). However, high C/N stress conditions induced *ATL31* expression similarly in the wild-type and *wrky33*, suggesting WRKY33 may not regulate *ATL31* expression in response to C/N stress (Reyes et al., 2015). Our work suggests WRKY33 also positively regulates *ATL31* expression in a pathway regulating hypocotyl elongation downstream of a selective auxin perception module that is activated by RN1 treatment (Supplemental Figure S4). Our results imply *WRKY33* also induces *ZAT10* expression, while *ATL31* appears to repress *ZAT10* expression, in a likely feedback mechanism leading to balanced regulation of further downstream signaling controlling hypocotyl elongation (Supplemental Figure S4). It is also tempting to speculate that these three genes, which are known to regulate stress responses, may additionally function in regulating the balance between stress resistance and growth. Many stress responses occur at the expense of growth and the existence of gene networks regulating such stress response/growth balance is perhaps not surprising.

These findings were made possible through the use of the selective molecule RN1 as a stimulator of hypocotyl elongation (Vain et al., 2019), highlighting the potential of chemical biology to unravel complex auxin signaling pathways and reveal molecular players in specific aspects of plant development. Previous successful chemical biology approaches have helped to dissect many details of complicated auxin biology, including its signaling, transport, and biosynthesis (Doyle et al., 2015b; Fukui and Hayashi, 2018; Torii et al., 2018). The effects of RN1 may be due to enhanced uptake compared to 2,4-D and/or a low-level, chronic 2,4-D release, as we previously demonstrated some 2,4-D is released via RN1 metabolism in planta (Vain et al., 2019) and it will be important to investigate this possibility in follow-up studies. However, the effects of direct, acute 2,4-D treatments on hypocotyl elongation and expression of the three candidate genes are distinct from those of RN1. RN1 is reminiscent of other 2,4-D analogs isolated previously, such as compounds 533 and 602 (Savaldi-Goldstein et al., 2008). Compound 602 also enhances hypocotyl elongation at concentrations not affecting root length and is thought to be hydrolyzed to release 2,4-D inside hypocotyl tissue, being more easily taken up than 2,4-D itself (Savaldi-Goldstein et al., 2008). Interestingly, compounds 533 and 602 both enhance TIR1-IAA7 interaction in pull-down assays and, similar to RN1, the double *tir1-1afb2-3* mutant is resistant to the effects of compound 602 on hypocotyl elongation (Savaldi-Goldstein et al., 2008). In summary, our work with RN1 strengthens the existing hypothesis that TIR1, AFB2, AFB5, and IAA7 are involved in auxin regulation of hypocotyl elongation, while adding evidence that activation of the *ZAT10*, *ATL31*, and *WRKY33* transcriptional network occurs downstream in the regulatory pathway.

## Conclusions

In this study, we took advantage of a synthetic small molecule inducing the degradation of the AUX/IAAs IAA3 and IAA7 in planta to reveal molecular players in auxin-induced hypocotyl elongation in Arabidopsis. Our results suggest the stress-related genes *ZAT10*, *ATL31*, and *WRKY33* take part in a common transcriptional regulatory network inducing hypocotyl elongation downstream of a specific auxin perception module in which the F-box receptors TIR1, AFB2, and AFB5 and degradation of the AUX/IAA transcriptional repressor IAA7 likely play important roles. This work paves the way for further studies detailing other molecular players in this network, such as the ARFs acting downstream of IAA7 degradation and upstream of our three candidate gene-encoded proteins, and elucidating the potential interactions of these proteins. It will also be of great interest to determine the players acting downstream of *ZAT10*, *ATL31*, and *WRKY33* and clarifying their specific actions in hypocotyl growth induction.

## Materials and methods

### Plant material

All experiments were performed with Arabidopsis (*A. thaliana*) Columbia-0 (Col-0) accession as the wild-type control, except for experiments using *shy2-2*, in which Ler accession was used. The Arabidopsis lines *axr1-30* (Hotton et al., 2011), *cul1-6* (Moon et al., 2007), *tir1-1* (Ruegger et al., 1998), *afb1-3*, *afb2-3*, *afb3-4*, *tir1-1afb2-3*, and *tir1-1afb1-3afb2-3afb3-4* (Parry et al., 2009), *afb5-5* (Prigge et al., 2016), *tir1-1afb5* (Gleason et al., 2011), *shy2-2/iaa3* (Reed et al., 1998; Tian and Reed, 1999), *axr2-1/iaa7* (Timpte et al., 1992; Nagpal et al., 2000), *zat10* (SALK_054092C; Alonso et al., 2003; Mittler et al., 2006), *35S::ZAT10* (Rossel et al., 2007), *wrky33* (GABI_324B11) and *WRKY33ox* (Birkenbihl et al., 2012; Kleinboelting et al., 2012), and *atl31-1* (GABI_746D08) and *35S::ATL31* (Sato et al., 2009; Kleinboelting et al., 2012) have been described previously. Homozygous *zat10wrky33*, *zat10atl31-1* and *atl31-1wrky33* double mutants were generated by crossing and genotyping (see Table 1 for primer sequences).

**Table 1.** Primers used in this study

RT-qPCR primers		
Gene	Forward primer sequence	Reverse primer sequence
*HAT2*	GAGAAGGAATCTCCGGAACC	CCGGAGTGATCTCGTCGT
*ZAT10*	TCACAAGGCAAGCCACCGTAAG	TTGTCGCCGACGAGGTTGAATG
*ATL31*	CAATCGGCGGTTCCTGTA	ATGCGACCTCGGGAATTTA
*WRKY33*	CTCTCCTTCCACTTGTTTCAGTCC	CTGTGGTTGGAGAAGCTAGAACG
*AT5G25760*	CTTAACTGCGACTCAGGGAATCTTC	AGGCGTGTATACATTTGTGCCATT
*AT1G13440*	TTGGTGACAACAGGTCAAGCA	AAACTTGTCGCTCAATGCAATC
*AT4G34270*	GGTTCCTCCTCTTGCGATT	ACAGTTGGTGCCTCATCTTC
*AT1G13320*	TAACGTGGCCAAAATGATGC	GTTCTCCACAACCGCTTGGT

Genotyping primers		

Mutant	Left primer	Right primer

*zat10*	TATTTTGTAAGGCGGCATCAG	AACGCGTTTGTAAAATATGTGG
*atl31-1*	ACATCACCGAACACTAAACCG	CTACTATTATCCGTGTCGGCG
*wrky33*	TTCACCAATCAGACGTGCAA	GTTTGGCTCCATTGTTCTGAC

Cloning primers		

Primer name	Sequence	

zat10prom_attB4	GGGGACAACTTTGTATAGAAAAGTTGAAAAATGCATAAGTTACTTG	
zat10prom_attB1	GGGGACTGCTTTTTTGTACAAACTTGTAAGTTAAAGATTCTGAGG	

Sequences of RT-qPCR, genotyping, and cloning primers are shown. For SALK (*zat10*) and GABI-Kat (*atl31-1*, *wrky33*) mutants, the left border primers LBb1.3 and o8474 were used, respectively.

### Plant and cell growth conditions

Arabidopsis seedlings were grown on vertical plates of growth medium containing 2.2-g·L^−1^ Murashige and Skoog (MS) medium, 0.5-g·L^−1^ MES, and 1% w/v sucrose at pH 5.6 with 0.7% w/v agar. Seedlings were grown for 5 d at 22°C with 16-h light per day. For the cDNA-AFLP experiment, Arabidopsis seedlings were grown in 25-mL liquid growth medium lacking agar at pH 5.9. The seedlings were grown under agitation at 95 rpm for 7 d at 21°C with 16-h light per day. Arabidopsis cell suspension culture medium contained 4.43-g·L^−1^ MS medium with minimal organics, 0.5-mg·L^−1^ NAA, 0.05-mg·L^−1^ kinetin, and 3% w/v sucrose at pH 5.7. The cultures were maintained under agitation at 130 rpm at 25°C in darkness and subcultured every 7 d by diluting 10-fold in fresh medium.

### Chemical treatments

RN1 (Chembridge ID 6389186; Vain et al., 2019) and 2,4-D stocks were dissolved in dimethyl sulfoxide (DMSO). The stock solutions were then diluted in liquid or solid growth medium for treatment at the indicated concentrations. Equal volumes of DMSO were used as mock treatment for control conditions.

### Hypocotyl and root length measurements

Hypocotyl and root length were measured in 5-d-old seedlings grown vertically on solid growth medium supplemented with 0.5–1-µM RN1 or 0.001–0.1-µM 2,4-D, using ImageJ software (https://imagej.nih.gov/ij/). Quantifications were made for 15–20 seedlings per sample for each of at least three biological replicates.

### GUS staining

Seedlings grown for 5 d on solid medium supplemented with 1-µM RN1 or 0.5-µM 2,4-D were fixed in 80% v/v acetone at −20°C for 20 min and then washed with distilled water. Seedlings were then incubated in 2 mM X-GlcA in β-glucuronidase (GUS) buffer at pH 7, infiltrated for 10 min in a vacuum and incubated in the dark at 37°C. The GUS buffer consisted of 0.1% v/v Triton X-100, 10-mM EDTA, 0.5-mM potassium ferrocyanide, and 0.5-mM potassium ferricyanide in 0.1 M phosphate buffer. Seedlings were then mounted in 50% v/v glycerol and observed on a Zeiss Axioplan microscope. GUS staining was performed on 5–10 seedlings per sample for each of at least three biological replicates and representative images were selected.

### RT-qPCR

Five-day-old seedlings grown vertically on solid growth medium were treated for 30 min with 50-µM RN1 or an equal volume of DMSO in liquid growth medium before extracting RNA. A PSB-D wild-type Arabidopsis cell culture (Arabidopsis Biological Resource Center stock: CCL84840) was grown after subculturing for 1 week and then divided into separate 10-mL samples for 24 h. Samples were then treated with 50-µM RN1, 2,4-D, NAA or an equal volume of DMSO before extracting RNA. Total RNA was extracted from 20 pooled whole seedlings per sample, or from each 10-mL cell culture sample, for each of at least three biological replicates, with the RNeasy Plant Kit (Qiagen) according to the manufacturer’s instructions. RQ1 RNase-free DNase (Promega) was used for the on-column DNase digestion step. cDNA was prepared from 1-µg total RNA with the iScript cDNA Synthesis Kit (Bio-Rad). RT-qPCR analyses on two technical replicates per sample were performed on a LightCycler 480 System (Roche Diagnostics) using SYBR Green I master mix (Roche Diagnostics) and the primers listed in Table 1. The genes *AT5G25760*, *AT1G13440*, *AT4G34270*, and *AT1G13320* were used as stable reference genes according to Doyle et al. (2015a). Two appropriate reference genes were selected using GeNorm (Biogazelle) for each set of experiments and relative expression levels were normalized to these two reference genes (Vandesompele et al., 2002).

### Pilot cDNA-AFLP experiment

Seven-day-old seedlings were treated with 1-µM RN1 or equivalent volume of DMSO, by directly adding the chemicals to the liquid growth medium. Two biological replicates were harvested after 30 min and 3 h of treatments. Total RNA was extracted using the RNeasy Plant Kit (Qiagen). For each sample, 2-µg total RNA was used. The pilot cDNA-AFLP-based transcriptomic profiling (20 primer combinations) was performed as described previously (Vuylsteke et al., 2007; Colling et al., 2013).

### RNAseq

RNA was extracted from Arabidopsis cell suspension culture samples (see “RT-qPCR” section) after treatment with 50-µM RN1 or 2,4-D for 30 min. RNA samples were processed by preparing a Trueseq RNAseq library (Illumina) and then sequenced at 30 million reads depth at 50-bp single read using Illumina HiSeq 2000 technology at GATC Biotech, Germany. Read quality control, filtering, mapping to the TAIR10 Arabidopsis genome and read counting were carried out using the Galaxy portal running on an internal server (http://galaxyproject.org/). Sequences were filtered and trimmed with the Filter FASTQ v1 and FASTQ Quality Trimmer v1 tools, respectively, using default settings (http://www.bioinformatics.babraham.ac.uk/projects/fastqc/). Reads were subsequently mapped to the TAIR10 version of the Arabidopsis genome using GSNAPv2, allowing a maximum of five mismatches. The concordantly paired reads that uniquely mapped to the genome were used for quantification on the gene level with htseq-count from the HTSeq python package. Data were normalized using TMM and common dispersion was then estimated using the conditional maximum-likelihood method implemented in edgeR64. Differentially expressed genes were defined by a 2-fold difference between samples with corrected *P* <0.05 at a false discovery rate <0.05.

### Cloning and generation of *ZAT10::GUS* reporter line

For the generation of promoter analysis lines of *ZAT10*, a promoter fragment was amplified by PCR using Pfu DNA Polymerase (Promega), in which the Gateway attB sites were added to the primers (see Table 1 for primer sequences). Then, the amplified sequences were purified and cloned into pDONR p4-p1r to build the entry vector. The *ZAT10* promoter in the entry vector was cloned together with the GUS reporter using the pEN-L2-S-L2 entry clone and the pK7m24GW destination binary vector using Gateway LR Clonase Plus (Invitrogen). The destination vector contained the terminator sequence from the 35S promoter and kanamycin resistance for the selection of transformants. All transgenic plants were generated by the floral dip method (Clough and Bent, 1998). The structure and sequence of entry and destination vectors are available online at https://gateway.psb.ugent.be.

### Statistical analyses

For all experiments, at least three biological replicates were performed, always on different days. The means and standard errors (se) of the means of all the biological replicates are displayed on charts. For parametric data, the Student’s *t* test was performed to compare two groups, while one-way analysis of variance followed by Tukey honestly significant difference post hoc tests were performed to compare three or more groups, for statistical differences. For nonparametric data, the Wilcoxon rank-sum test was performed to compare two groups, while Kruskal–Wallis one-way analysis of variance followed by Wilcoxon rank-sum tests were performed to compare three or more groups, for statistical differences.

### Accession numbers

The Arabidopsis Genome Initiative numbers for the genes featured in this study are as follows. *AFB1*: *AT4G03190*; *AFB2*: *AT3G26810*; *AFB3*: *AT1G12820*; *AFB5*: *AT5G49980*; *ATL31*: *AT5G27420*; *AUF1*: *AT1G78100*; *AUX2-27/IAA5*: *AT1G15580*; *AXR1*: *AT1G05180*; *AXR2/IAA7*: *AT3G23050*; *AXR5/IAA1*: *AT4G14560*; *CUL1*: *AT4G02570*; *GH3.2*: *AT4G37390*; *HAT2*: *AT5G47370*; *SHY2/IAA3*: *AT1G04240*; *TIR1*: *AT3G62980*; *WRKY33*: *AT2G38470*; *ZAT10*: *AT1G27730*. The RNAseq datasets generated in this study are available in the ArrayExpress database at EMBL-EBI under accession number E-MTAB-7761 (http://www.ebi.ac.uk/arrayexpress/experiments/E-MTAB-7761).

We thank VINNOVA and the Knut and Alice Wallenberg Foundation for financial support to Umeå Plant Science Centre.

## Supplemental data

The following materials are available in the online version of this article.

**Supplemental Figure S1.** 2,4-D weakly increases hypocotyl length while strongly decreasing root length.

**Supplemental Figure S2.** RN1-induced gene expression in *ZAT10*-, *ATL31*-, and *WRKY33*-affected lines.

**Supplemental Figure S3.** RN1-induced hypocotyl elongation in *ZAT10*-, *ATL31*-, and *WRKY33*-affected lines.

**Supplemental Figure S4.** Model of postulated signaling pathway regulating hypocotyl elongation in Arabidopsis light-grown seedlings, induced by treatment with RN1.

**Supplemental Table S1.** cDNA-AFLP-based transcript proﬁling after RN1 treatment.

**Supplemental Table S2.** Genes differentially regulated by RN1 and 2,4-D treatments determined by RNAseq-based transcript proﬁling.

## Funding 

This work in particular was funded by the Plant Fellows fellowship program (A.Rig.), the Knut and Alice Wallenberg Foundation “ShapeSystems” grant no. 2012.0050 (S.M.D., S.Ro.), Stiftelsen Olle Engkvist Byggmästare grant no. 185 595 (S.Ra.), VINNOVA/Vetenskapsrådet grant no. VR 2013-4632 (T.V.), the Belgian Science Policy organization for a postdoctoral fellowship (A.Rit.), and the Research Foundation Flanders for a postdoctoral fellowship (L.P.) and for research grant no. 1507013N (A.Rit., A.G., L.P.).

*Conflict of interest statement*. None declared.

## Supplementary Material

kiab269_Supplementary_Data

## Notes

These authors contributed equally to the article (A.Rig., S.M.D.).

A.Rig., L.P., and S.Ro. conceived the project; A.Rig., S.M.D., A.Rit., S.Ra., T.V., and J.A.O’B. performed the experiments; A.G., L.P., and S.Ro. supervised the research; A.Rig., S.M.D., and S.Ro. wrote the article with contributions of all the authors.

The author responsible for distribution of materials integral to the findings presented in this article in accordance with the policy described in the Instructions for Authors (https://academic.oup.com/plphys/pages/general-instructions) is: Stéphanie Robert (stephanie.robert@slu.se).
