# Supplementary material for: A network of stress-related genes regulates hypocotyl elongation downstream of selective auxin perception
Source: Plant Physiol. 2021 Jun 12;187(1):430–45. doi: 10.1093/plphys/kiab269 (PMC8418399; doi:10.1093/plphys/kiab269)
Supplement: kiab269_Supplementary_Data [file kiab269_supplementary_data.zip › pp.00316.2021-s02.pdf]

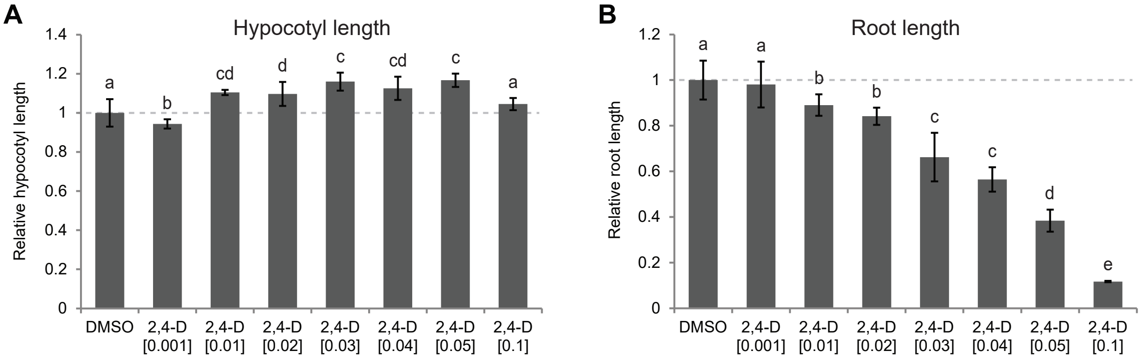

Supplemental Figure S1. 2,4-D weakly increases hypocotyl length while strongly decreasing root length. A-B, Hypocotyl length (A) and root length (B) relative to DMSO treatment in 5-day-old wild type seedlings treated with 2,4-D for 5 d. Means of at least 3 biological replicates are shown, horizontal dashed lines represent DMSO and error bars represent SE of the mean. Different letters indicate statistical differences according to the Tukey-Kramer HSD test or Wilcoxon rank-sum test at $\alpha = 0.05$. Concentrations in square brackets are in μM.

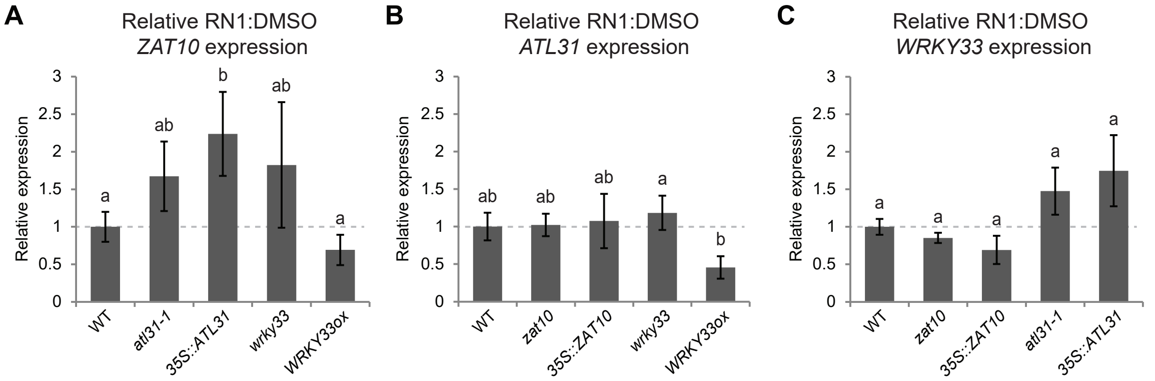

Supplemental Figure S2. RN1-induced gene expression in *ZAT10*-, *ATL31*- and *WRKY33*-affected lines. A-C, Expression of *ZAT10* (A), *ATL31* (B) and *WRKY33* (C) relative to DMSO treatment after 30 min of 50 µM RN1 treatment in 5-day-old mutant and over-expressor lines, relative to that in wild type (WT). Gene expression was analyzed in mutant and over-expressor lines of *ZAT10* (*zat10* and *35S::ZAT10*), *ATL31* (*atl31-1* and *35S::ATL31*) and *WRKY33* (*wrky33* and *WRKY33ox*). Means of at least 3 biological replicates are shown, horizontal dashed lines represent WT and error bars represent SE of the mean. Different letters indicate statistical differences according to the Tukey-Kramer HSD test or Wilcoxon rank-sum test at α = 0.05.

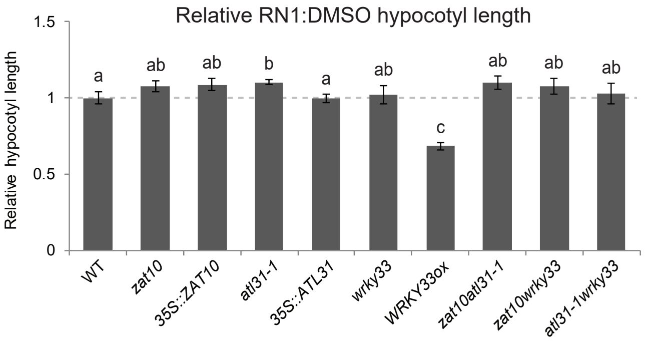

Supplemental Figure S3. RN1-induced hypocotyl elongation in *ZAT10*-, *ATL31*- and *WRKY33*-affected lines. Hypocotyl length relative to DMSO treatment after 5 d of 1 μM RN1 treatment in 5-day-old mutant and over-expressor lines of *ZAT10*, *ATL31* and *WRKY33*, relative to that in wild type (WT). Hypocotyl length was analyzed in mutant and over-expressor lines of *ZAT10* (*zat10* and *35S::ZAT10*), *ATL31* (*atl31-1* and *35S::ATL31*) and *WRKY33* (*wrky33* and *WRKY33ox*) as well as in the homozygous double mutants *zat10atl31-1, zat10wrky33* and *atl31-1wrky33.* Means of at least 3 biological replicates are shown, horizontal dashed line represents WT and error bars represent SE of the mean. Different letters indicate statistical differences according to the Wilcoxon rank-sum test at $\alpha = 0.05$.

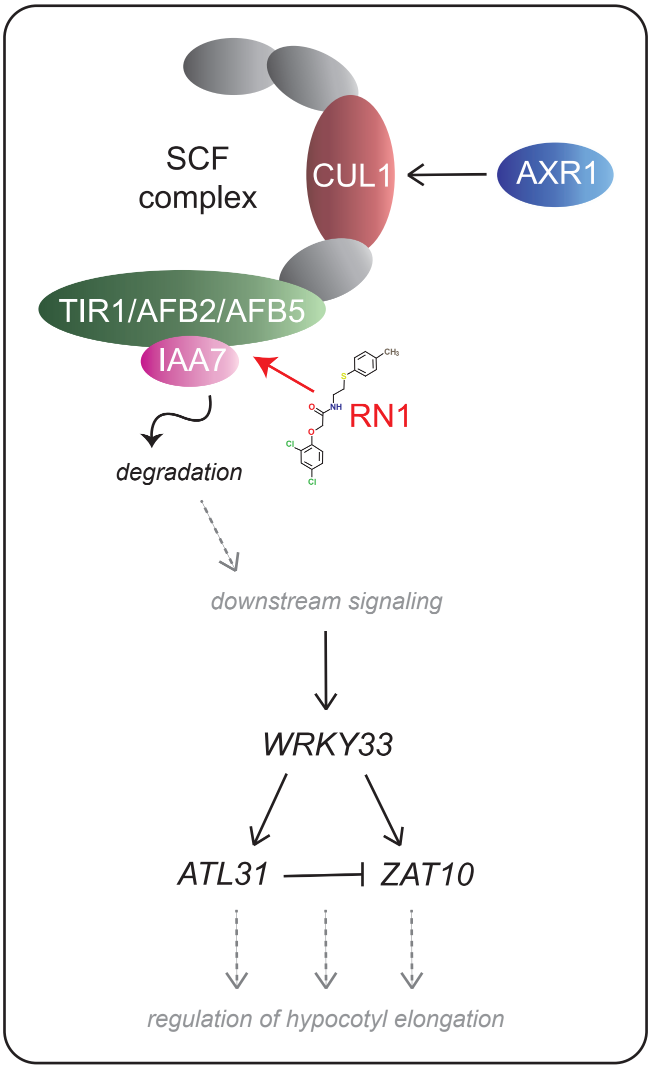

Supplemental Figure S4. Model of postulated signaling pathway regulating hypocotyl elongation in Arabidopsis light-grown seedlings, induced by treatment with RN1. We hypothesize that RN1 selectively promotes the interaction of the TIR1, AFB2 and AFB5 F-box receptors with the AUX/IAA transcriptional repressor IAA7, which leads to IAA7 degradation and subsequent activation of downstream signaling. *WRKY33* expression is induced downstream of this pathway and acts upstream of *ATL31* and *ZAT10*, in turn inducing their expression. Meanwhile, *ATL31* acts upstream of *ZAT10*, suppressing its expression. Hypocotyl elongation is regulated downstream of this signaling pathway.

Supplemental Table S1. cDNA-AFLP-based transcript profiling after RN1 treatment. List of the genes differentially regulated by 30 min or 3 h of 1 μM RN1 treatment compared to DMSO treatment in 7-day-old Arabidopsis seedlings, determined by cDNA-AFLP, including the Arabidopsis Genome Initiative (AGI) number. Up- and down-regulation are indicated by + and – respectively.

AGI number	30 minutes	3 hours	Encoded protein name	Brief description
AT5G57220	+		CYTOCHROME P450 - FAMILY 81 - SUBFAMILY F - POLYPEPTIDE 2 (CYP81F2)	Member of CYP81F, involved in glucosinolate metabolism.
AT2G38020	-		EMB258, MANGLED, VACUOLELESS 1 (VCL1)	Necessary for proper vacuole formation and morphogenesis in Arabidopsis.
AT3G21250	+		MULTIDRUG RESISTANCE-ASSOCIATED PROTEIN 6 (MRP6), ATP-BINDING CASSETTE C8 (ABCC8)	Member of MRP subfamily.
AT3G55840	+			Hs1pro-1 protein.
AT3G52180	+		PTPKIS1, DUAL-SPECIFICITY PROTEIN PHOSPHATASE 4 (DSP4), STARCH-EXCESS 4 (SEX4)	Encodes a plant-specific glucan phosphatase with a noncatalytic carbohydrate-binding module and a dual specificity protein phosphatase domain.
AT1G27730	+		SALT TOLERANCE ZINC FINGER (STZ), ZINC FINGER OF ARABIDOPSIS THALIANA 10 (ZAT10)	Related to Cys2/His2-type zinc-finger proteins found in higher plants. Acts as a transcriptional repressor.
AT5G45340	+		CYTOCHROME P450 - FAMILY 707 - SUBFAMILY A - POLYPEPTIDE 3 (CYP707A3)	Encodes a protein with ABA 8'-hydroxylase activity, involved in ABA catabolism.
AT3G16700	+			Fumarylacetoacetate (FAA) hydrolase family.
AT5G41130	+			Esterase/lipase/thioesterase family protein.
AT2G26980	-		CBL-INTERACTING PROTEIN KINASE 3 (CIPK3), SNF1-RELATED PROTEIN KINASE 3.17 (SNRK3.17)	Encodes a serine-threonine protein kinase whose expression increases in response to abscisic acid, cold, drought, high salt and wounding conditions.
AT4G12030	+		BILE ACID TRANSPORTER 5 (BAT5), BILE ACID:SODIUM SYMPORTER FAMILY PROTEIN 5 (BASS5)	Required for the biosynthesis of methionine-derived glucosinolates. Involved in the transport of 2-keto acids between chloroplasts and the cytosol.
AT3G23550	+		DETOXIFICATION 18 (DTX18)	MATE efflux family protein.
AT4G31700	+		RIBOSOMAL PROTEIN S6 (RPS6/RPS6A)	Encodes a putative ribosomal protein S6 (RPS6A).
AT3G14420		+	GLYCOLATE OXIDASE 1 (GOX1)	Encodes a glycolate oxidase that modulates reactive oxygen species-mediated signal transduction during nonhost resistance.
